# Evaluating preventive measures for the zoonotic transmission of swine influenza A variant at agricultural fairs in the United States: a mathematical modeling study

**DOI:** 10.3389/fvets.2025.1590156

**Published:** 2025-05-30

**Authors:** Dana C. Pittman Ratterree, Sapna Chitlapilly Dass, Martial L. Ndeffo-Mbah

**Affiliations:** ^1^Department of Veterinary Integrative Biosciences, College of Veterinary Medicine and Biomedical Sciences, Texas A&M University, College Station, TX, United States; ^2^Department of Animal Science, College of Agriculture and Life Sciences, Texas A&M University, College Station, TX, United States; ^3^Department of Epidemiology and Biostatistics, School of Public Health, Texas A&M University, College Station, TX, United States

**Keywords:** agricultural fair, mathematical modeling, prevention, swine influenza, zoonotic events

## Abstract

**Introduction:**

Swine exhibitions at agricultural fairs serve as unique environments where humans and pigs interact, and swine influenza A can spill over. As agricultural fairs present a substantial risk for zoonotic influenza outbreaks and potential pandemics, it is paramount to identify efficient preventive measures for mitigating the risk of variant influenza A transmission from pigs to humans at swine exhibitions.

**Methods:**

We developed a mathematical model of swine influenza A variant transmission at agricultural fairs. We fitted the model to empirical data of a 2011 zoonotic outbreak at a Pennsylvania agricultural fair. We used the fitted model to simulate and evaluate the impact of various control strategies, including preventive measures such as shortening the exhibition duration, enhanced biosecurity, pre-fair testing of pigs, and quarantine of sick animals.

**Results:**

The impact of control strategies was shown to vary substantially between preventive measures. Shortening the length of the exhibition to 3 days generated the lowest prevalence of disease in pigs and humans. Increased biosecurity measures reduced the risk and size of swine influenza outbreaks among pigs and humans during the exhibition period. Due to the majority of pigs experiencing asymptomatic infections, case identification and quarantining of sick pigs did not significantly reduce the infection prevalence.

**Conclusion:**

Shortening the duration of swine exhibitions combined with enhanced biosecurity measures was shown to be the most effective method for preventing zoonotic transmission of swine influenza during agricultural fairs in the US. The study provides additional evidence on the potential benefits of shortened swine exhibitions.

## 1 Introduction

Pigs are a critical reservoir for zoonotic influenza as it is a common respiratory disease in domestic swine (*Sus scrofa domesticus*) (1–4). More specifically, influenza A in swine (IAV-S) has 3 major serotypes circulating among agricultural swine populations: H1N1, H1N2, and H3N2 (4–6). Variant influenza refers to human infections caused by swine-origin viruses (7, 8). A primary risk factor for zoonotic swine influenza is contact with pigs through occupational exposure at swine production facilities or exposure at swine exhibitions hosted at agricultural fairs (7, 8). Since 2010, the United States Centers for Disease Control and Prevention (CDC) has recorded 546 confirmed swine-origin variant influenza A cases in the United States (7, 8). The majority of these cases (321) came from the 2011–2012 flu season of which 90% were associated with swine exposure at agricultural fairs across the United States (7–9). These spillover events of swine-origin influenza A have a significant pandemic potential as demonstrated by the 2009 H1N1 swine influenza pandemic (10, 11).

Annually, more than 3,000 agricultural fairs are held in the United States over the summer, many including some form of a swine exhibition (10). These events draw agricultural club participants and their pigs from across states to compete for prizes and showcase livestock (10). With hundreds of thousands of attendees, these fairs enable direct human-pig contact, creating opportunities for viral transmission of swine influenza and other swine-related pathogens (9, 10). Subclinical influenza infections are predominant in pigs, making detection of the virus before the exhibition or identifying cases while the event is being held difficult (12–14). Agricultural fairs have been the leading source of zoonotic swine influenza exposure for the general public in the United States (9, 10). Epidemiological investigations of swine influenza spillover events, linked to agricultural fairs, have been recorded in Ohio, Michigan, Maryland, and Pennsylvania (15–21).

In response to the surge in zoonotic cases of variant influenza A associated with agricultural fair swine exhibitions during the 2011–2012 flu season, the Swine Exhibitions Zoonotic Influenza Working Group was established in 2012 (22–24). This group is composed of organizations and stakeholders such as the United States Department of Agriculture (USDA), the United States Centers for Disease Control and Prevention (CDC), the National Pork Board, and the National Future Farmers of America Organization (FFA) (23). The recommendations proposed by this working group can be split into two: prevention measures targeted at reducing infection among swine, and those targeted at human infection risk. In humans, the recommendations focus on increasing the availability of hand washing stations, discouraging toys or food from being brought into the exhibition hall, and preventing interaction with visibly sick pigs (22, 25). The primary preventive measure recommended for reducing transmission in the swine population focuses on the isolation of sick pigs, enhanced biosecurity through planning, and shortening the duration of swine exhibition to 72 h (14, 22).

In this study, we investigated measures to minimize the transmission of influenza A variant from pigs to humans at agricultural fairs. We develop a mathematical model of a zoonotic swine influenza outbreak at an agricultural fair that explicitly accounts for both clinical and subclinical infection among pigs by adapting previous models (15, 26). We fitted our model to a 2011 zoonotic outbreak at a Pennsylvania agricultural fair. We used the fitted model to investigate the impact of preventive measures, recommended by the Swine Exhibitions Zoonotic Influenza Working Group, to reduce the risk of swine-to-swine and swine-to-human transmission of a swine influenza A variant during swine exhibitions. These measures include shortening the exhibition’s duration, implementing enhanced biosecurity, pre-fair testing, and quarantining of pigs showing clinical signs.

## 2 Methods

### 2.1 Epidemiological model

We developed a mathematical model for the transmission of variant influenza A among pigs and between pigs and humans in an agricultural fair setting. We expand upon previous dynamic population models for the transmission of the H3N2 influenza A variant during agricultural fairs (15, 26). We expanded the simple SEIR (Susceptible-Exposed-Infected-Recovered) model to include a second infected compartment denoted as A for “asymptomatic” to account for subclinical infections in pigs. Human disease dynamics follows an SEIR (Susceptible-Exposed-Infected-Recovered) model. The following ordinary differential equations describe the model dynamics:


$$\frac{{\mathrm{dS}}_{s}}{\mathrm{dt}}=-\beta \times S_{s}\times (\frac{I_{s}+A_{s}}{N_{s}})$$

$$\frac{{\mathrm{dE}}_{s}}{\mathrm{dt}}=\beta \times S_{s}\times (\frac{I_{s}+A_{s}}{N_{s}})-\kappa_{s}E_{s}$$

$$\frac{{\mathrm{dA}}_{s}}{\mathrm{dt}}=\kappa_{s}E_{s}\delta -\gamma_{s}A_{s}$$

$$\frac{{\mathrm{dI}}_{s}}{\mathrm{dt}}=\kappa_{s}E_{s}(1-\delta )-\gamma_{s}I_{s}$$

$$\frac{{\mathrm{dR}}_{s}}{\mathrm{dt}}=\gamma_{s}A_{s}+\gamma_{s}I_{s}$$

$$\frac{{\mathrm{dS}}_{h}}{\mathrm{dt}}=-C\times P\times S_{h}\times (\frac{I_{s}+A_{s}}{N_{s}})$$

$$\frac{{\mathrm{dE}}_{h}}{\mathrm{dt}}=C\times P\times S_{h}\times (\frac{I_{s}+A_{s}}{N_{s}})-\kappa_{h}E_{h}$$

$$\frac{{\mathrm{dI}}_{h}}{\mathrm{dt}}=\kappa_{h}E_{h}-\gamma_{h}I_{h}$$

$$\frac{{\mathrm{dR}}_{h}}{\mathrm{dt}}=\gamma_{h}I_{h}$$

Where *S_h_*, *E_h_*, *I_h_*, *R_h_* represent susceptible, exposed, infectious, and recovered humans, respectively, and *S_s_*, *E_s_*, *A_s_*, *I_s_*, *R_s_* represent susceptible, exposed, asymptomatic (subclinical) infectious, infectious, and recovered swine, respectively. The human model parameters include: the probability of transmission per minute of contact with swine is *P*, duration of contact in minutes with infected swine is *C*, total number of swine is with *N_s_*, the rate exposed humans progress to infected is *κ_h_* (1/incubation), the rate of human recovery is *γ_h_* (1/duration of illness). The swine model parameters include: swine-to-swine transmission rate of the infection is *β*, the recovery rate of swine is *γ_s_*, and the proportion of asymptomatic (subclinical) swine infections is *δ*. We fitted out model to data from a 2011 zoonotic outbreak at a Pennsylvania agricultural fair (15, 16), see Supplementary materials. We used this Pennsylvania fair outbreak as our baseline “no control” scenario. Parameter values are listed in Table 1. The descriptive flow diagram of our model is provided in Supplementary Figure 1.

**Table 1:** Baseline model parameters.

Parameter symbol	Parameter description	Value	Source
–	Total susceptible exhibitor population with swine contact*	90	(15)
–	Total susceptible attendee population with swine contact*	14,910	(15, 16)
–	Duration of the swine exhibition*	9 days	(15, 16)
*C*	Contact duration*	60 min (exhibitor) 5 min (attendee)	(15)
*P*	Probability of transmission	0.0035 (exhibitor) 0.002 (attendee)	Fitted
*N_s_*	Total number of exhibited swine*	208	(15, 16)
1/κ_h_	Incubation period*	2 days	(15, 16)
1/γ_h_	Duration of infection in humans	5 days	(37)
β	Swine-to-swine transmission rate	R_0_/γ_s_	(3)
1/κ_s_	Incubation period in pigs	2 days	(38)
1/γ_s_	Duration of infection in swine	5 days	(38, 39)
R_0_	Reproduction number	Varied	(3)
δ	Proportion of asymptomatic (subclinical) infections in swine	0.83	(12)
1/*θ*	Duration from detection to quarantine	1 day	Assumed
χ	Proportion of pigs identified to have clinical signs	Varied (0.2–1)	–

*Obtained directly from epidemiological investigations of the 2011 zoonotic outbreaks.

The parameter values were mostly adapted from a 2011 zoonotic outbreak at a Pennsylvania agricultural fair (15, 16).

Our model has two modes of transmission: swine-to-swine and swine-human. The parameter *β* for swine-to-swine transmission is defined as the product of the basic reproduction number (R_0_) and the recovery rate (27). Here we assume pigs with clinical signs and subclinical infections are equally likely to transmit the virus. This assumption is supported by observations at agricultural fairs in Ohio where fairs with asymptomatic pigs had comparable infection prevalence among pigs (12). Additionally, our model assumed new infections in pigs only come from exposure to infected pigs. Though pigs can be infected with influenza from humans, the contact duration is limited compared to the hours of prolonged exposure from cohabitating in the exhibition barn (12–15). Moreover, our modeling framework does not incorporate sustained human-to-human transmission of the variant influenza A. This is consistent with existing literature, which emphasizes the role of direct contact with infected swine as a primary risk factor for zoonotic transmission (9, 15, 20). Our model accounts for rapid viral transmission induced by high levels of environment contamination observed by Lauterbach et al. (28). With an R_0_ baseline value of 6, more than 60% of the pigs become infected by the end of the fair (Supplementary Figure 2).

To simulate quarantining of infectious animals we modify the pig population model by adding a *Q* compartment for quarantined pigs with clinical signs. The ordinary differential equations for pigs now become:


$$\frac{{\mathrm{dS}}_{s}}{\mathrm{dt}}=-\beta \times S_{s}\times (\frac{I_{s}+A_{s}}{N_{s}})$$

$$\frac{{\mathrm{dE}}_{s}}{\mathrm{dt}}=\beta \times S_{s}\times (\frac{I_{s}+A_{s}}{N_{s}})-\kappa_{s}E$$

$$\frac{{\mathrm{dA}}_{s}}{\mathrm{dt}}=\kappa_{s}\mathrm{E\delta}-\gamma_{s}A_{s}$$

$$\frac{{\mathrm{dI}}_{s}}{\mathrm{dt}}=\kappa_{s}E(1-\delta )-\chi \theta_{s}I_{s}-(1-\chi )\gamma_{s}I_{s}$$

$$\frac{{\mathrm{dR}}_{s}}{\mathrm{dt}}=\gamma_{s}A_{s}+(1-\chi )\gamma_{s}I_{s}$$

$$\frac{\mathrm{dQ}}{\mathrm{dt}}=\chi \theta_{s}I_{s}$$

Where the proportion of pigs identified with clinical signs is *χ* and the quarantine rate is *θ*. Where 1/θ is the average duration between case identification and isolation. We assume once a pig is quarantined it no longer contributes to transmission.

### 2.2 Scenario analysis

To estimate the potential size of human cases and prevalence in pigs, we employed a stochastic version of our model. We used the *τ*-leap methodology for the stochastic simulation with a Poisson distribution as described by Keeling and Rohani (29). We ran 10,000 simulations for each preventive measure to capture the range of possible outcomes for prevalence in the pig population and cumulative infection count in humans by summing the club member and attendee populations. The deterministic and stochastic models were constructed in the integrative development environment Spyder (version 6.0.3, Spyder Website Contributors; 2024). Model fitting was performed in MATLAB R2023a (version: 9.14.0, Natick, Massachusetts: The MathWorks Inc., 2023). Data visualization and calculations of central tendency for the *τ*-leap simulation results were performed in RStudio (version: 2024.9.0.375, Boston, Massachusetts: Posit Software).

Our baseline scenario has no intervention, where there are 5 initially infected pigs, the R_0_ value equals 6, and the exhibition duration is 9 days (15). Our model also assumes 17% of the infected pigs have clinical signs based on a surveillance study by Bowman et al. at agricultural fairs in Ohio (12). To simulate preventive strategies recommended by the Swine Exhibitions Zoonotic Influenza Working Group, we varied different parameter values to simulate each recommendation. We consider 7 exhibition durations ranging from 3 to 9 days. To investigate the impact of biosecurity on outbreak risk, we assumed that enhanced biosecurity will reduce R_0_ value by 33% or 50% (here resulting in a R_0_ value of 4, or 3), and poor biosecurity will increase R_0_ value by 33% or 50% (here resulting in a R_0_ value of 8, or 9). In the quarantine of sick pigs model, we vary the proportion of identified pigs with clinical signs (*χ*) between 0.2–1 simulating a range of abilities for exhibitors to identify pigs with influenza-like illnesses. We also consider a scenario where there are twice as many symptomatic pigs (*δ* = 35%) to determine how the prevalence of symptomatic animals impacts the ability and the efficacy of quarantine for reducing outbreak risk. For pre-fair testing, we assume that all pigs are tested at the start of the fair. We considered a 60 and 80% test sensitivity which correlates to identifying and isolating 60/80% of initially infected pigs arriving at the exhibition. At baseline, we assume that there were 5 infected pigs at the start of the fair. With an 80% test sensitivity, 80% of initially infected pigs will be identified and isolated before the start of the fair and on average only 1 infected pig will remain in the fair. With 60% sensitivity, only 2 initially infected pigs will remain in the fair. The recommended exhibition duration by the swine working group is 72 h (3 days), but exhibitions such as the one investigated by Wong et al. last as long as 9 days (14–16, 22).

Additionally, we consider 12 combinations of preventive measures. Scenarios 1–8 combine enhanced biosecurity (50 and 33% reduction of transmission risk) and shortened exhibition durations (ED = 3, 4, 5, 7) (Table 2). Scenarios 9–12 combine pre-fair testing (Tests = 60, 80%), quarantine of sick animals, and enhanced biosecurity (50 and 33% reduction of transmission risk) (Table 2). In these scenarios, we assumed that all infected pigs with clinical signs were identified and quarantined (*χ* = 1).

**Table 2:** Intervention strategies.

Label	Description	Outcome
Baseline	R_0_ = 6, ED = 9 days	Swine and human infection count
Scenario 1	50% reduction of transmission risk (R_0_ = 3), ED = 3 days	Swine and human infection count
Scenario 2	50% reduction of transmission risk (R_0_ = 3), ED = 4 days	Swine and human infection count
Scenario 3	50% reduction of transmission risk (R_0_ = 3), ED = 5 days	Swine and human infection count
Scenario 4	50% reduction of transmission risk (R_0_ = 3), ED = 7 days	Swine and human infection count
Scenario 5	33% reduction of transmission risk (R_0_ = 4), ED = 3 days	Swine and human infection count
Scenario 6	33% reduction of transmission risk (R_0_ = 4), ED = 4 days	Swine and human infection count
Scenario 7	33% reduction of transmission risk (R_0_ = 4), ED = 5 days	Swine and human infection count
Scenario 8	33% reduction of transmission risk (R_0_ = 4), ED = 7 days	Swine and human infection count
Pre-fair testing, enhanced biosecurity, and quarantine of sick pigs		
Scenario 9	50% reduction of transmission risk (R_0_ = 3), Tests = 80%, ED = 9 days, θ = 1/day	Swine and human infection count
Scenario 10	33% reduction of transmission risk (R_0_ = 4), Tests = 80%, ED = 9 days, θ = 1/day	Swine and human infection count
Scenario 11	50% reduction of transmission risk (R_0_ = 3), Tests = 60%, ED = 9 days, θ = 1/day	Swine and human infection count
Scenario 12	33% reduction of transmission risk (R_0_ = 3), Tests = 60%, ED = 9 days, θ = 1/day	Swine and human infection count

ED: Exhibition duration, Tests: Test sensitivity, θ is the rate pigs are quarantined.

## 3 Results

### 3.1 Exhibition duration

**Figure 1:**
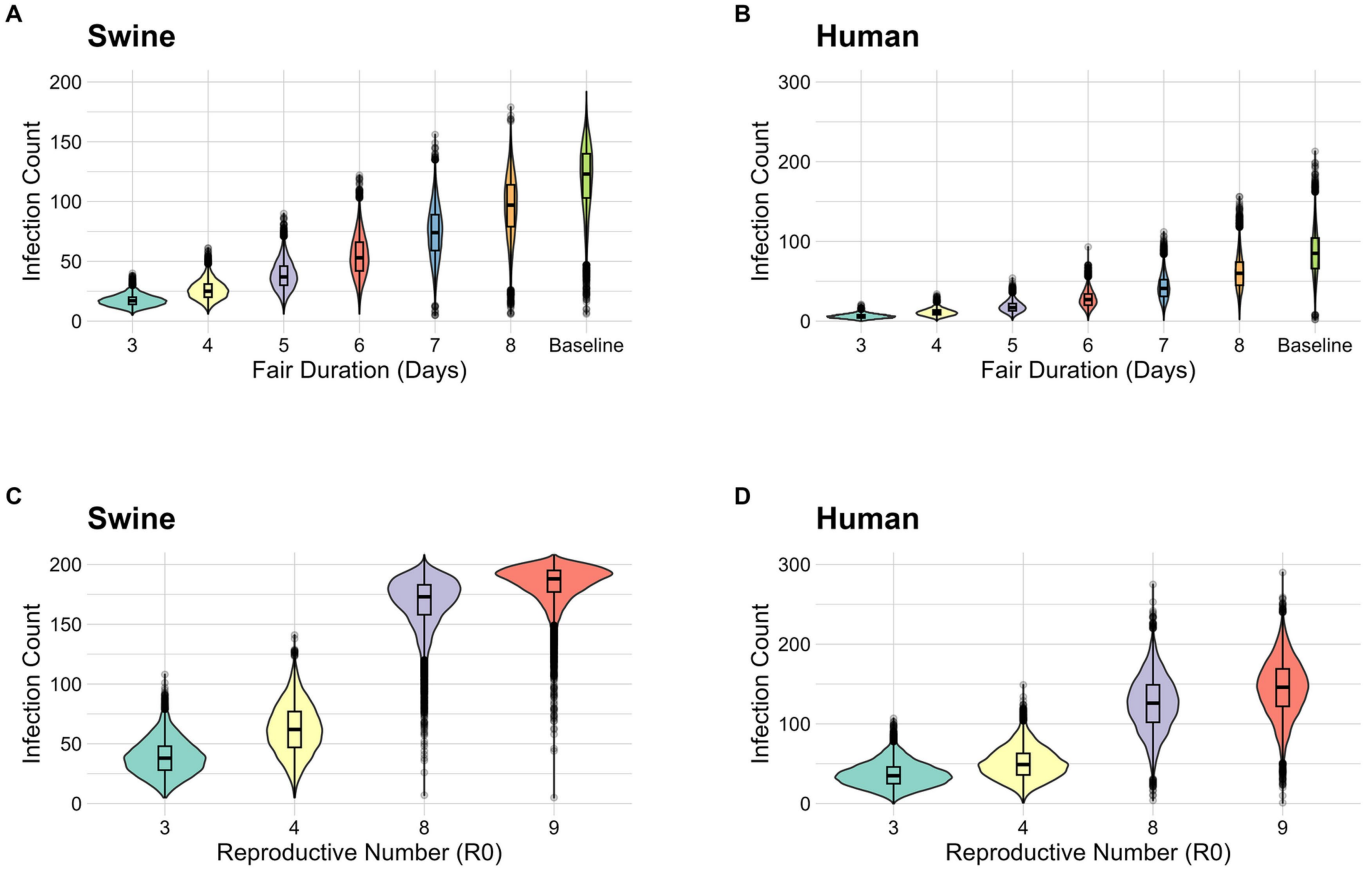
**(A)** Stochastic simulation of the number of infections in pigs (both latent and infectious) by fair duration. **(B)** Stochastic simulation of the number of human infections by fair duration. **(C)** Stochastic simulation of the number of infections in pigs (both latent and infectious) by R_0_. **(D)** Stochastic simulation of the number of human infections by R_0._ Because the number of pigs exhibited is 208_,_ the y-axis for pigs is limited to 200.

The longer the exhibition duration, the larger the range of possible infection prevalence and larger outbreaks were observed (Figures 1A,B). Shorter exhibition durations generated simulations with a lower number of infections in both pigs and humans. In the scenarios when the exhibition duration is 3, 6, and 9 days the mean number of infected pigs was 17.1 (Interquartile Range (IQR): 14–20), 54.1 (IQR: 42–66), and 120.1 (IQR: 103–140) (Figure 1A and Supplementary Table 1), respectively. Infected pigs account for both latent (E_s_) and infectious (A_s_, I_s_) animals during the period of the fair. Among both exhibitors and general attendees, the cumulative case count in humans were 6.14 (IQR: 4–8), 27.9 (IQR: 20–34), and 85.8 people (IQR: 66–104.15), respectively (Figure 1B and Supplementary Table 1). Shortening the duration of the exhibition from 9 days to 3 days reduced the mean number of infected pigs by 85.8% and the mean number of infected humans by 92.8%.

### 3.2 Biosecurity

We varied the R_0_ value from 4 to 9 to consider different biosecurity levels (Figure 1). In a poor biosecurity setting, where the baseline transmission risk is increased by 50% (R_0_ = 9) the mean number of infected pigs is 183.5 (IQR: 177–195) and the mean case count in humans is 144.6 (IQR: 122–169) (Supplementary Table 2 and Figures 1C,D). Whereas, in an enhanced biosecurity setting, where the baseline transmission risk is reduced by 50% (R_0_ = 3) the mean case count in pigs decreased to 38.6 (IQR: 28–48) and the mean cumulative case count in humans was 36.5 (IQR: 25–46) (Supplementary Table 2 and Figures 1C,D). Higher values of R_0_ (poor biosecurity) produced higher mean values for both the number of infected pigs and the cumulative case count in humans. By improving biosecurity, the mean number of infected pigs was reduced by 79.0% and the mean number of infected humans was reduced by 74.8%.

### 3.3 Quarantine of pigs with clinical signs

To determine the impact of quarantine and the proportion of pigs with clinical signs on disease transmission risk we added a quarantine (Qs) compartment to our model and varied the proportion of pigs with clinical signs identified from 20% to 100%. At baseline, we assumed that 17% of infected pigs developed clinical signs (Is) that can be identified by exhibitors or staff veterinarians. When the proportion of sick pigs identified was 20% the mean number of infected pigs was 116.5 (IQR: 99–137) and the mean cumulative case count in humans was 81.9 (IQR: 62–101). Increasing the proportion identified to 100% decreased the mean case count in pigs to 108.8 (IQR: 91–129) and the mean case count in humans to 73.3 (IQR: 56–91) (Supplementary Table 3 and Supplementary Figures 3A,B). When the proportion of sick pigs identified increased from 20 to 100%, the mean number of infected pigs reduced by 6.6% and the number of infected humans decreased by 10.5%. Increasing the proportion of pigs with clinical signs to 35% did not qualitatively change the results (Supplementary Table 3 and Supplementary Figures 3C,D).

### 3.4 Pre-fair testing

In pre-fair testing, we assumed a test sensitivity of 80% (60%), meaning that 4 (3) of the 5 initially infected pigs were identified and isolated before the swine exhibition. When there was one initially infected pig (80% sensitivity), the mean number of infected pigs was 37.1 (IQR: 12–57) and the mean cumulative case count in humans was 21 (IQR: 6–32) (Supplementary Table 4 and Supplementary Figure 4). With two initially infected pigs (60% sensitivity), the mean number of infected pigs was 64.5 (IQR: 41–89) and the mean cumulative case count in humans was 39.8 (IQR: 22–55) (Supplementary Table 4 and Supplementary Figure 4). With only one initially infected pig, 13.8% of our simulations produced no additional infections and 10.67% of simulations produced no human infections. Increasing the number initially infected to two decreased the number of simulations with no additional pig infections or human infections to 2.06 and 1.23%, respectively.

### 3.5 Combination strategies

**Figure 2:**
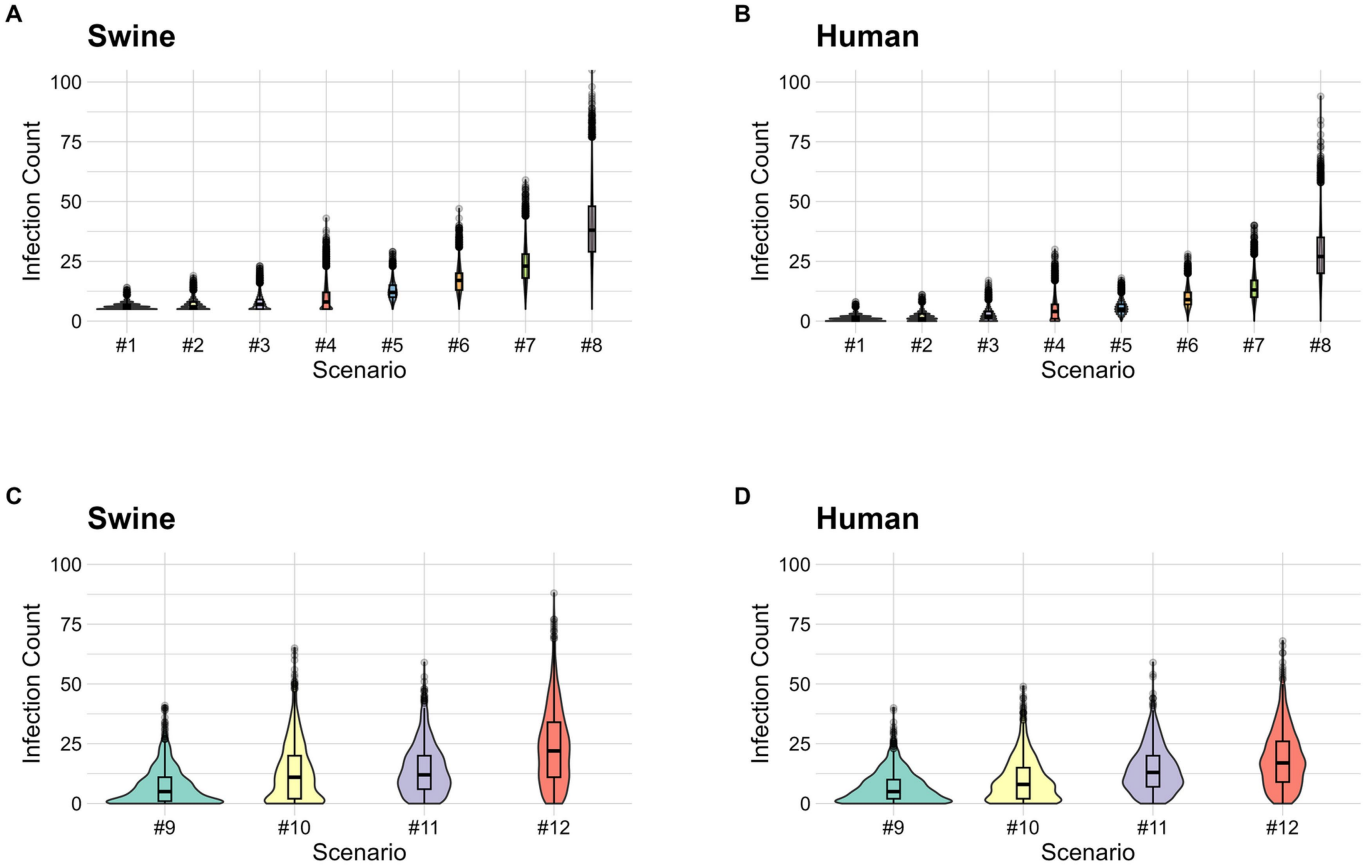
**(A)** Stochastic simulation of the number of infections in pigs (both latent and infectious) for combined exhibition duration and biosecurity scenarios. **(B)** Stochastic simulation of the number of human infections for combined exhibition duration and biosecurity scenarios. **(C)** Stochastic simulation of the number of infections in pigs (both latent and infectious) for combined pre-fair testing, improved biosecurity, and quarantine of sick animal scenarios. **(D)** Stochastic simulation of the number of human infections for combined pre-fair testing, improved biosecurity, and quarantine of sick animal scenarios. The simulated scenarios are defined as follow: scenario #1 (50% reduction of transmission risk from enhanced biosecurity and 3 days exhibition duration), scenario #2 (50% reduction of transmission risk from enhanced biosecurity and 4 days exhibition duration), scenario #3 (50% reduction of transmission risk from enhanced biosecurity and 5 days exhibition duration), scenario #4 (50% reduction of transmission risk from enhanced biosecurity and 7 days exhibition duration), scenario #5 (33% reduction of transmission risk from enhanced biosecurity and 3 days exhibition duration), scenario #6 (33% reduction of transmission risk from enhanced biosecurity and 4 days exhibition duration), scenario #7 (33% reduction of transmission risk from enhanced biosecurity and 5 days exhibition duration), scenario #8 (33% reduction of transmission risk from enhanced biosecurity and 7 days exhibition duration), scenario #9 (50% reduction of transmission risk, 9 days exhibition duration, 80% testing efficacy, quarantine of all clinical cases 1 day after onset of clinical signs), scenario #10 (33% reduction of transmission risk, 9 days exhibition duration, 80% testing efficacy, quarantine of all clinical cases 1 day after onset of clinical signs), scenario #11 (50% reduction of transmission risk, 9 days exhibition duration, 60% testing efficacy, quarantine of all clinical cases 1 day after onset of clinical signs), scenario #12 (33% reduction of transmission risk, 9 days exhibition duration, 60% testing efficacy, quarantine of all clinical cases 1 day after onset of clinical signs).

The first set of scenarios combines R_0_ equal to 3 or 4 and exhibition durations of 3, 4, 5, and 7 days (Table 2). Scenario one had the lowest mean number of infections in both pigs and humans; the mean number of infections in pigs was 6.1 (IQR: 5–7) and the mean cumulative case count in humans was 1.1 (IQR: 0–2) (Supplementary Table 5 and Figures 2A,B). Additionally, we combined pre-fair testing and enhanced biosecurity to identify which combination produced the lowest prevalence in pigs and cumulative case count in humans (Table 2). Among this combination of preventive measures, scenario 9 produced the lowest mean number of infections in pigs of 7.1 (IQR: 1–11) and a mean case count of 7 (IQR: 2–10) in humans (Supplementary Table 6 and Figures 2C,D).

## 4 Discussion

This study employs computational modeling to evaluate the effectiveness of control strategies aimed at reducing both swine-to-swine and swine-to-human transmission of a swine influenza A variant in a swine exhibition setting. To achieve this objective, we adapted the structure of previous models for variant influenza transmission at agricultural fairs to account for both clinical and subclinical infections in pigs (15, 26) and fitted our model to empirical data of a 2011 zoonotic outbreak during an agricultural fair in Pennsylvania. In addition to introducing separate compartments for infected classes in pigs, we also developed a model where pigs with clinical signs were moved into the quarantine compartment. We assessed four preventive strategies recommended by the Swine Exhibitions Zoonotic Influenza Working Group using our model (22). The results indicate that the effectiveness of control strategies was shown to vary substantially with the preventive measures.

Among the four preventive measures we investigated, shortening the duration of the exhibition was the most effective individual preventive measure. Our result is consistent with observations of lower disease prevalence in pigs when the exhibition is shortened to 72 h compared with week-long events (14). It should be noted that a potential unintended consequence of shortening the duration of swine exhibitions may be an increased geographic spread of the disease as exhibitors may be able to attend more fairs, during a shorter time period, to increase their odds of winning prizes (10, 30, 31). Future work should investigate the feasibility of such an outcome. The present model shows that identification and quarantine of sick animals alone is ineffective for reducing disease transmission due to the high percentage of subclinical influenza infections among pigs. While it could reduce human contact with sick animals and lower spillover risks, it does not significantly reduce infections in humans or pigs compared to shorter fair durations or enhanced biosecurity. For quarantining to work, exhibitors need training to spot illness and have access to quarantine facilities. The model suggests quarantine should be used alongside more effective measures, like reducing exhibition duration. In addition to enhanced biosecurity and shortening the duration of the exhibition, agricultural fairs should encourage record-keeping practices in case there is an outbreak investigation so pigs that are moved between fairs and the origin of the animal are documented (32, 33). Individual pigs often attend multiple agricultural exhibitions across states each year, increasing opportunities for intrastate and interstate mixing and spread of influenza A viruses (10, 31, 32, 34).

Due to limited high-quality data on zoonotic influenza outbreaks at agricultural fairs, current investigations often fail to distinguish between cases among general attendees and exhibitors or provide details on fair activities, attendance, and human-swine interactions—key factors for model parameterization. Exhibitors face higher infection risks due to closer, prolonged contact with pigs but may also have greater immunity from previous exposures compared to general attendees (15, 16, 19, 21, 35). Models of swine influenza transmission should account for these differences to better estimate zoonotic risks at fairs. Accurate parameterization will require future studies to provide detailed information on case incidence and risk activities among general fair attendees and exhibitors, and infection prevalence among show pigs. It is paramount to reduce the risk of transmission at agricultural fairs, as hundreds to thousands of people who do not regularly interact with pigs can become exposed at these events (12, 30, 36). Furthermore, swine exhibitions allow animals from various farms to commingle over days, and it only takes one infected pig to produce an outbreak (12, 34, 36).

For simplicity, our model was parameterized using point value estimates. This does not account for the direct impact of epidemiological parameter values’ variability on the model’s predictions. To mitigate the impact of this assumption on our results, we undertook several scenario analyses, such as investigating the impact of varying the value of the basic reproduction number. Because of our model’s underlying simplicity, we anticipate that adding variability to parameter values would not alter the qualitative nature of our results and would only have a marginal quantitative impact. Furthermore, we assumed that subclinical and clinical disease in pigs are equally infectious. Though this assumption may cause the model to overestimate the contribution of subclinically infected pigs to disease transmission, we anticipate its impact to be marginal on our results, as empirical studies have observed comparable disease prevalence between fairs with a high number of subclinical pigs and fairs with higher numbers of clinical infections (12). Another limitation caused by the simplicity of the model is the homogeneous mixing assumption both among pigs and between pigs and humans. To address this issue, future work should use an agent-based modeling approach to account for heterogeneity in contact that has the potential to play a pivotal role in pathogen spread during agricultural fairs. However, parameterizing such a model will require extensive data on pig-pig and pig-human contacts during agricultural fairs, which may not be currently available.

But focusing only on disease transmission during the agricultural fair, our model underestimates the burden of an influenza A variant outbreak from hog exhibitions. Our model does not account for the fact that latent and infectious pigs who leave the fair can infect other pigs or humans once they return to their farms. Though our analysis focuses primarily on agricultural fairs, we cannot lose track of the fact that backyard livestock poses a significant threat to biosecurity, particularly in the context of increasing Influenza A outbreaks. A substantial proportion of swine exhibited at fairs originates from backyard operations, creating a potential pathway for disease transmission. Given the rising prevalence of various Influenza A strains, this study would provide context for implementing enhanced biocontainment measures in backyard livestock settings and raise awareness about the implications for agricultural fairs.

## Data Availability

The original contributions presented in the study are included in the article/Supplementary material, further inquiries can be directed to the corresponding author. The code can be found at: https://github.com/dana-pittman/SEAIR_Model.
