# Supplementary material for: Evaluating preventive measures for the zoonotic transmission of swine influenza A variant at agricultural fairs in the United States: a mathematical modeling study
Source: Front Vet Sci. 2025 May 30;12:1590156. doi: 10.3389/fvets.2025.1590156 (PMC12162892; doi:10.3389/fvets.2025.1590156)
Supplement: Supplementary file 1 [file Data_Sheet_1.docx]

Supplementary Material

# Supplementary Figures

**
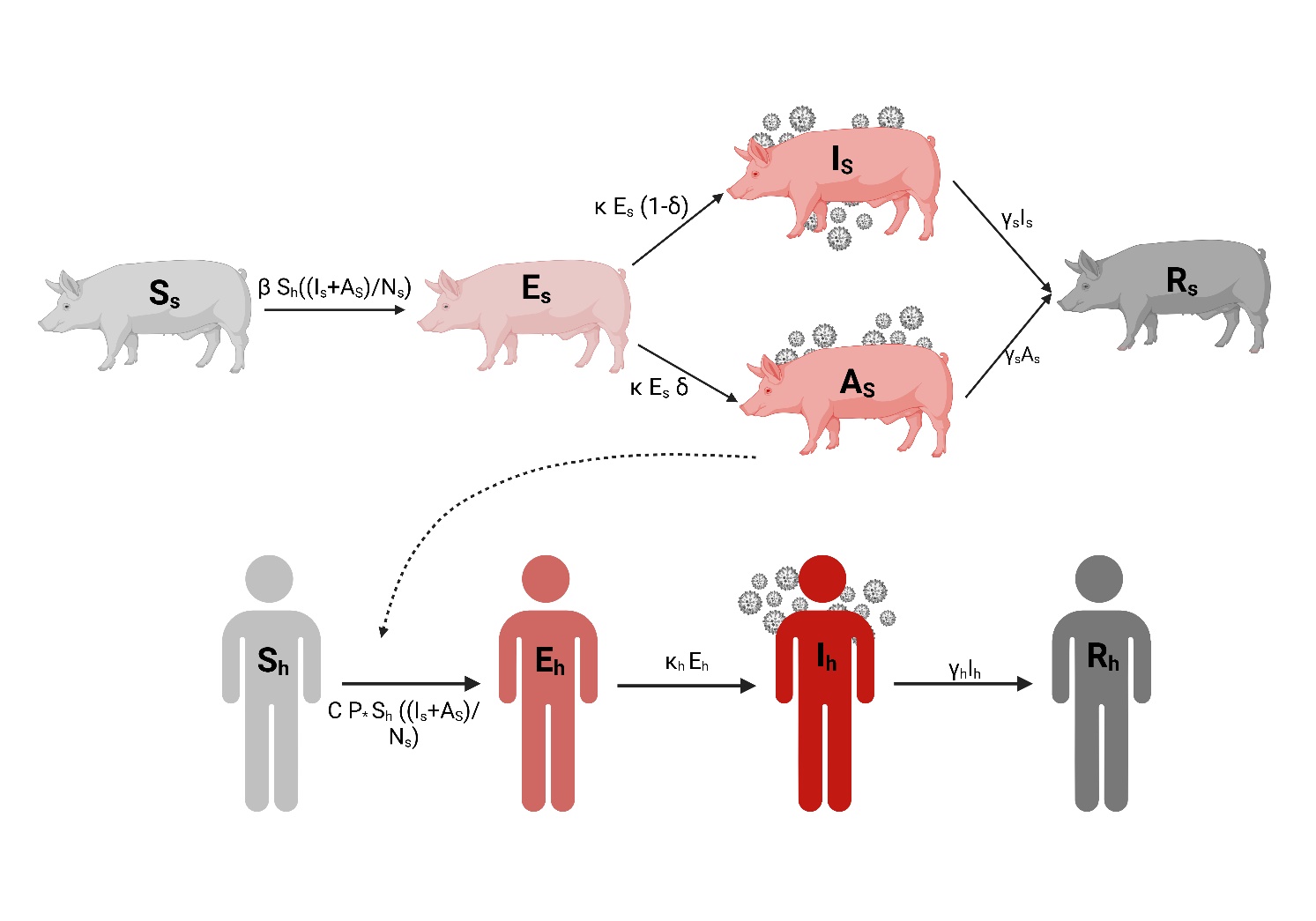
**

**Supplementary Figure 1.** Compartmental diagram for the transmission of influenza A variant between swine and human populations. Created in BioRender. Pittman, D. (2025) <https://BioRender.com/rf6gdsl>


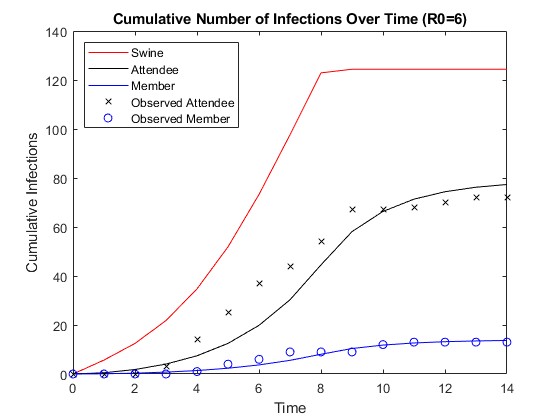


**Supplementary Figure 2.** Model Fitting results using maximum likelihood estimation when R_0_ is 6.

**
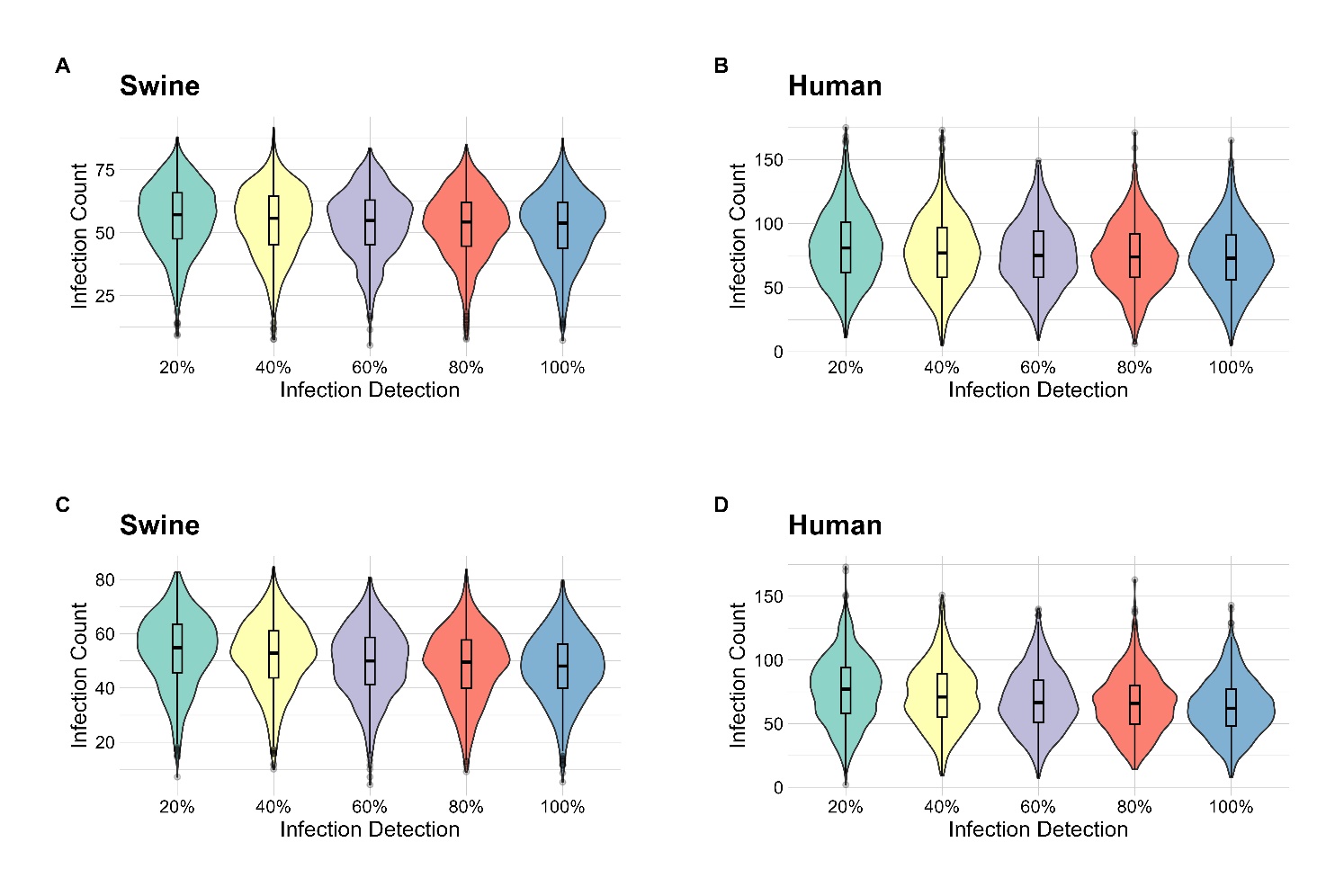
Supplementary Figure 3.** a) Stochastic simulation of the swine infection prevalence, when 17% of pigs have clinical signs, under varying proportions of symptomatic pigs identified and quarantined. b) Stochastic simulation of the number of human infections, when 17% of pigs have clinical signs, under varying proportions of symptomatic pigs identified and quarantined. c) Stochastic simulation of the swine infection prevalence, when 35% of pigs have clinical signs, under varying proportions of symptomatic pigs identified and quarantined. d) Stochastic simulation of the number of human infections, when 35% of pigs have clinical signs, under varying proportions of symptomatic pigs identified and quarantined.

**
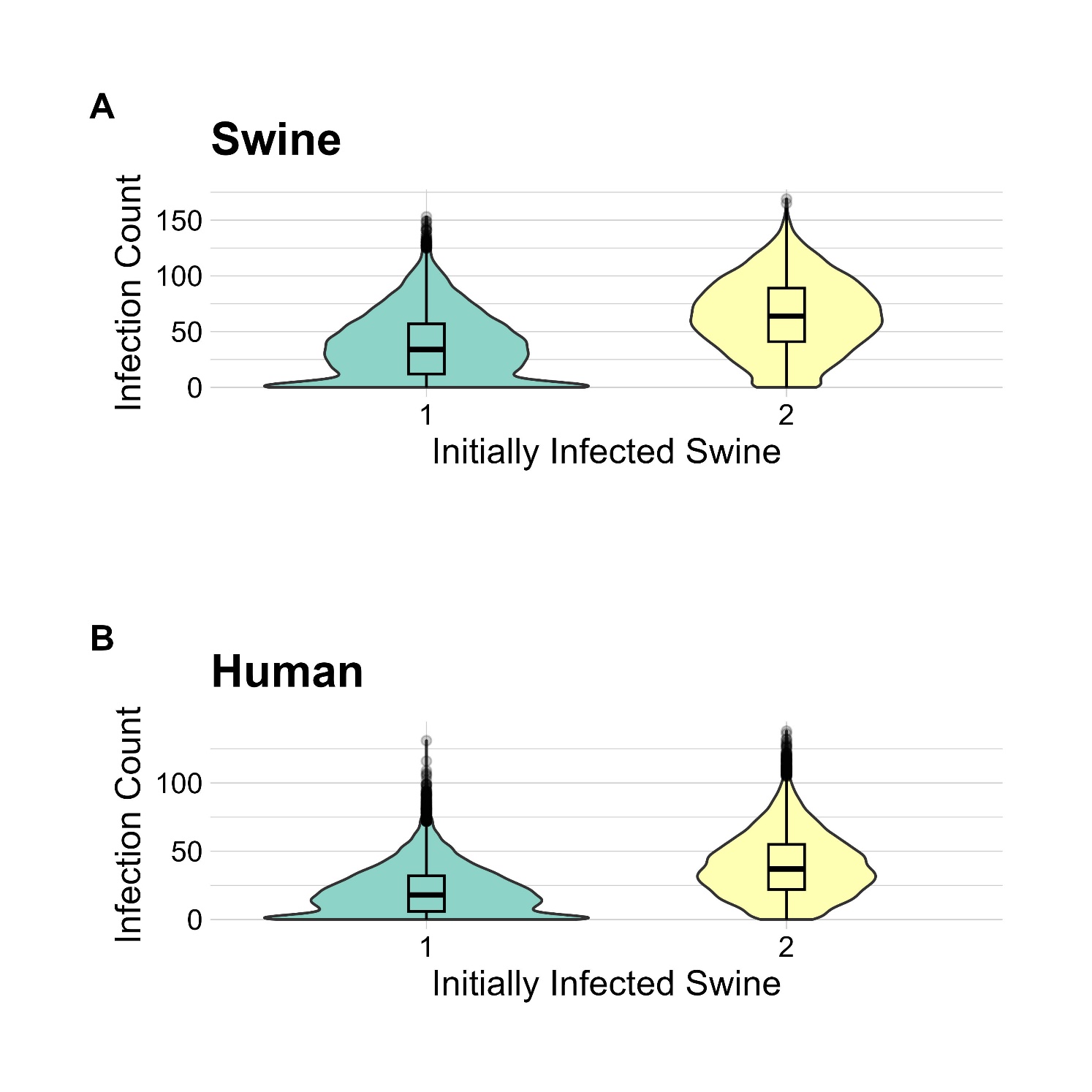
Supplementary Figure 4.** a) Stochastic simulation of the swine infection prevalence when pre-fair testing reduces the number of initially infected swine. b) Stochastic simulation of the number of human infections when pre-fair testing reduces the number of initially infected swine.

# Supplementary Tables

**Supplementary Table 1.** Summary statistics of stochastic simulations for each fair duration.

| Population | Duration | Mean Case Count | Quartile 1 Case Count | Quartile 3 Case Count |
| --- | --- | --- | --- | --- |
| Swine | 3 | 17.1 | 14 | 20 |
| Swine | 4 | 26.0 | 20 | 31 |
| Swine | 5 | 38.2 | 30 | 46 |
| Swine | 6 | 54.1 | 42 | 66 |
| Swine | 7 | 74.0 | 59 | 89 |
| Swine | 8 | 96.0 | 79 | 114 |
| Swine | Baseline | 120.1 | 103 | 140 |
| Human | 3 | 6.14 | 4 | 8 |
| Human | 4 | 10.9 | 8 | 14 |
| Human | 5 | 18.0 | 13 | 22 |
| Human | 6 | 27.9 | 20 | 34 |
| Human | 7 | 42.1 | 31 | 52 |
| Human | 8 | 60.7 | 45 | 74 |
| Human | Baseline | 85.8 | 66 | 104.25 |

**Supplementary Table 2.** Summary statistics of stochastic simulations for biosecurity.

| Population | R_0_ | Mean Case Count | Quartile 1 Case Count | Quartile 2 Case Count |
| --- | --- | --- | --- | --- |
| Swine | 3 | 38.6 | 28 | 48 |
| Swine | 4 | 62.2 | 47 | 77 |
| Swine | 8 | 167.9 | 158 | 183 |
| Swine | 9 | 183.5 | 177 | 195 |
| Human | 3 | 36.5 | 25 | 46 |
| Human | 4 | 50.5 | 36 | 63 |
| Human | 8 | 125.6 | 102 | 149 |
| Human | 9 | 162.5 | 139 | 187 |

**Supplementary Table 3.** Summary statistics of stochastic simulations for quarantine of pigs with clinical signs.

| Population | Clinical Signs (%) | Detection (%) | Mean Case Count | Quartile 1 Case Count | Quartile 3 Case Count |
| --- | --- | --- | --- | --- | --- |
| Pig | 17 | 20 | 116.5 | 99 | 137 |
| Pig | 17 | 40 | 112.9 | 94 | 134.25 |
| Pig | 17 | 60 | 111.5 | 94 | 131 |
| Pig | 17 | 80 | 110.4 | 93 | 129 |
| Pig | 17 | 100 | 108.8 | 91 | 129 |
| Pig | 35 | 20 | 111.7 | 95 | 132 |
| Pig | 35 | 40 | 108.0 | 91 | 127 |
| Pig | 35 | 60 | 102.9 | 86 | 122 |
| Pig | 35 | 80 | 100.7 | 83 | 120 |
| Pig | 35 | 100 | 99.0 | 83 | 117 |
| Human | 17 | 20 | 81.9 | 62 | 101 |
| Human | 17 | 40 | 77.9 | 58 | 97 |
| Human | 17 | 60 | 76.2 | 58 | 94 |
| Human | 17 | 80 | 74.6 | 58 | 92 |
| Human | 17 | 100 | 73.3 | 56 | 91 |
| Human | 35 | 20 | 76.6 | 58 | 94 |
| Human | 35 | 40 | 72.4 | 55 | 89 |
| Human | 35 | 60 | 67.9 | 51 | 84 |
| Human | 35 | 80 | 65.5 | 49.75 | 80 |
| Human | 35 | 100 | 63.0 | 48 | 77 |

**Supplementary Table 4.** Summary statistics of stochastic simulations for pre-fair testing.

| Population | As | Mean Case Count | Quartile 1 Case Count | Quartile 3 Case Count |
| --- | --- | --- | --- | --- |
| Swine | 1 | 37.1 | 12 | 57 |
| Swine | 2 | 64.5 | 41 | 89 |
| Human | 1 | 21.1 | 6 | 32 |
| Human | 2 | 39.8 | 22 | 55 |

†As: Initial number of infected swine.

**Supplementary Table 5.** Summary statistics of stochastic simulations for improved biosecurity and fair duration

| Population | Scenario | Mean Case Count | Quartile 1 Case Count | Quartile 3 Case Count |
| --- | --- | --- | --- | --- |
| Swine | 1 | 6.1 | 5 | 7 |
| Swine | 2 | 6.8 | 5 | 8 |
| Swine | 3 | 7.5 | 5 | 9 |
| Swine | 4 | 9.7 | 5 | 12 |
| Swine | 5 | 12.6 | 10 | 15 |
| Swine | 6 | 17.2 | 13 | 20 |
| Swine | 7 | 23.2 | 18 | 28 |
| Swine | 8 | 39.4 | 29 | 48 |
| Human | 1 | 1.1 | 0 | 2 |
| Human | 2 | 1.7 | 0 | 3 |
| Human | 3 | 2.5 | 1 | 4 |
| Human | 4 | 4.6 | 1 | 7 |
| Human | 5 | 5.7 | 4 | 7 |
| Human | 6 | 9.4 | 7 | 12 |
| Human | 7 | 14.0 | 10 | 17 |
| Human | 8 | 28.0 | 20 | 35 |

**Supplementary Table 6.** Summary statistics of stochastic simulations for combined pre-fair testing, improved biosecurity, and quarantine of sick animal scenarios.

| Population | Scenario | Mean Case Count | Quartile 1 Case Count | Quartile 3 Case Count |
| --- | --- | --- | --- | --- |
| Pig | 9 | 7.1 | 1 | 11 |
| Pig | 10 | 13.1 | 2 | 20 |
| Pig | 11 | 14.3 | 6 | 20 |
| Pig | 12 | 23.7 | 11 | 34 |
| Human | 9 | 7.0 | 2 | 10 |
| Human | 10 | 10.0 | 2 | 15 |
| Human | 11 | 14.2 | 7 | 20 |
| Human | 12 | 18.8 | 9 | 26 |
